# Sleep Disturbance and Strain Among Caregivers of Persons Living With Dementia

**DOI:** 10.3389/fnagi.2021.734382

**Published:** 2022-02-03

**Authors:** Zainab Toteh Osakwe, Charles Senteio, Omonigho Michael Bubu, Chinedu Obioha, Arlener D. Turner, Sujata Thawani, Rose Saint Fleur-Calixte, Girardin Jean-Louis

**Affiliations:** ^1^College of Nursing and Public Health, Adelphi University, Garden City, NY, United States; ^2^School of Communication and Information, Rutgers University, New Brunswick, NJ, United States; ^3^Department of Population Health, New York University Grossman School of Medicine, New York, NY, United States; ^4^Department of Psychiatry, New York University Grossman School of Medicine, New York, NY, United States; ^5^Department of Psychiatry and Behavioral Sciences and Neurology, University of Miami, Miami, FL, United States; ^6^Department of Neurology, New York University Grossman School of Medicine, New York, NY, United States; ^7^SUNY Downstate Medical Center, Brooklyn, NY, United States

**Keywords:** caregivers, dementia, sleep disparities, strain, NHATS

## Abstract

**Objective:**

The study objective was to examine predictors of sleep disturbance and strain among caregivers of persons living with dementia (PLWD).

**Methods:**

This cross-sectional study utilized a sample of community-dwelling older adults and their family caregivers drawn from the 2017 National Health and Aging Trends Study and National Study of Caregiving. Multivariable logistic regression was used to assess the association between caregiver and PLWD characteristics and a composite measure of caregiving strain. High caregiving strain was defined as a total score of ≥ 5 on the 6 caregiving strain items (e.g., emotional difficulty, no time for self). We used multivariable proportional odds models to examine predictors of caregiver sleep-related outcomes (trouble falling back to sleep and interrupted sleep), after adjusting for other caregiver and PLWD factors.

**Results:**

Of the 1,142 family caregivers, 65.2% were female, 15% were Black, and 14% were Hispanic. Average age was 60 years old. Female caregivers were more likely to report high level of strain compared to male caregivers (OR: 2.61, 95% CI = 1.56, 4.39). Compared to non-Hispanic Whites, non-Hispanic Black and Hispanic caregivers had reduced odds of reporting greater trouble falling back asleep [OR = 0.55, CI (0.36, 0.82) and OR = 0.56, CI (0.34, 0.91), respectively]. The odds of reporting greater trouble falling back asleep was significantly greater among caregivers with high blood pressure vs. caregivers without high blood pressure [OR = 1.62, CI (1.12, 2.33)].

**Conclusion:**

In this cross-sectional study, caregivers with greater sleep difficulty (trouble falling back asleep) were more likely to report having high blood pressure. We found no racial/ethnic differences in interrupted sleep among caregivers to PLWD. These results suggest that interventions to improve sleep among caregivers to PLWD may decrease poor cardiovascular outcomes in this group.

## Introduction

In the United States, 70% of persons living with dementia (PLWD) live in the community (Hebert et al., 2013; Alzheimer’s Association, 2019) and rely on family and unpaid caregivers (familycaregivers) to avoid or delay nursing home placement (Kasper et al., 2015; Black et al., 2019). About 16.3 million caregivers, provide 18.6 billion unpaid hours of dementia caregiving (Gehrman et al., 2018). These caregivers provide the majority of long-term care and support the complex care need of PLWD aging in their own homes (Freedman and Spillman, 2014; Roth et al., 2015; Wolff et al., 2016). Family caregivers assist with personal care, and health related care needs, in addition to providing social support (Wolff et al., 2016; Burgdorf et al., 2020). Caregiving to PLWD is particularly demanding associated with high caregiver burden (Brodaty et al., 2014) and can place a toll on caregivers’ own mental and physical health (Schulz and Martire, 2004; Brodaty et al., 2014; Roth et al., 2018). Increase in the frequency of behavioral and psychiatric symptoms problems (e.g., agitation, restlessness, wandering) in PLWD or the provision of ongoing assistance with health care needs increase caregiver strain (Jennings et al., 2015; Vick et al., 2019). These caregiving demands are complex and are known to impact the sleep quality of caregivers negatively (Leggett et al., 2018; Liang et al., 2020).

Caregivers for PLWD also report a range of sleep problems in their care recipients, including difficulty falling asleep and nighttime awakenings (McCurry et al., 1999; Tractenberg et al., 2005; Rose et al., 2011). Sleep disturbances in the PLWD that they care for can result in the caregivers themselves experiencing symptoms of sleep disturbance (Ornstein and Gaugler, 2012; Gehrman et al., 2018; Liang et al., 2020). A recent systematic review and meta -analysis of 35 studies found that caregivers for PLWD report shorter and poorer sleep quality compared to non-caregiving adults (Gao et al., 2019). Previous investigators indicate that sleep disturbances among caregivers to PLWD also negatively impacts caregivers, and affect health (Mills et al., 2009) and quality of life (QOL) (Liang et al., 2020; Mather et al., 2020). Indeed, sleep problems are among the most challenging concerns reported by caregivers of PLWD, and are related to increased caregiver burden and likelihood of nursing home placement of the care recipient (Donaldson et al., 1998).

Available data suggest that disparities in the caregiving burden of PLWD exist. Blacks and Hispanics have a higher prevalence of disability in later life, suggesting the possibility of a disproportionate burden on caregivers of PLWD (Skolarus et al., 2017; Cook and Cohen, 2018). Prior work also points to disparities in sleep problems, with Black and Hispanic individuals experiencing more sleep disturbance than non-Hispanic whites (Ownby et al., 2010; Williams et al., 2016). These disparities may contribute to greater burdens for family and unpaid caregivers of PLWD in minority communities. Indeed, prior studies have found that relative to white caregivers, Black and Hispanic caregivers report heavier caregiving demands and assist older adults with greater functional impairment (Cook and Cohen, 2018; Badana et al., 2019; Reinhard et al., 2019; Fabius et al., 2020).

Given the possible disproportionate burden in Black and Hispanic populations, we used linked, nationally representative surveys of Medicare beneficiaries and their family caregivers to explore racial/ethnic differences in sleep and strain among caregivers to PLWD. Findings are relevant to ongoing efforts by researchers and clinicians to improve the health of family caregivers to PLWD.

## Materials and Methods

### Participants and Study Design

Data were obtained from the 2017 National Health and Aging Trends Study (NHATS) and the linked National Survey of Caregiving (NSOC). Conducted since 2011, NHATS is an ongoing nationally representative survey of Medicare beneficiaries 65 years of age and older in the United States, over sampling older ages and Black beneficiaries (DeMatteis et al., 2016). The NHATS survey is conducted annually in-person with participants and/or proxy respondents covering topics that include functional status, medical care, and cognition (Montaquila et al., 2012). The NSOC is a telephone survey of caregivers who assist NHATS participants. To be eligible for NSOC, a family or unpaid non-family caregiver must have provided the NHATS participant who resides in the community help with self-care, mobility or household activities (Kasper et al., 2013).

This study focused on NHATS participants with dementia living in the community and their caregivers who responded to NSOC. We excluded persons living in nursing homes due to our focus on PLWD aging at home. We applied NHATS criteria to identify dementia status (Galvin et al., 2005; Kasper et al., 2013). NHATS has a validated algorithm to determine whether a participants has probable dementia based on any of the following indicators: (1) self- or proxy report of being told by a doctor that the participant has dementia or Alzheimer’s disease, (2) a score indicating probable dementia on the validated AD8 Dementia Screening Interview administered to proxy respondents, and (3) cognitive test results (Kasper et al., 2013). NHATS participants were classified as having probable dementia if they met criteria for impairment in at least two cognitive domains and as having possible dementia if they had impairment in one cognitive domain (Kasper et al., 2013). Similar to prior research, NHATS participants who did not meet criteria for possible or probable dementia were classified as having no dementia (Amjad et al., 2016).

### Measures

The outcome variables of interest were caregiver-reported strain and sleep-related disturbances derived from caregiver-reported measures.

#### Caregiver Strain

Caregiver strain was measured with a composite variable (range: 0–9), constructed from six items, consistent with prior approaches (Wolff et al., 2016, 2018; Vick et al., 2019). Similar to prior work using NSOC data, high caregiving strain was defined based on a total score pf 5 or greater on the six caregiving strain items (Wolff et al., 2016; Vick et al., 2019). The 6-item caregiver strain measure was computed by summing three items that capture emotional, physical, financial difficulty of caregiving, and three items reflective of the negative aspects of caregiving (having no time for oneself, being overwhelmed, and being exhausted). For questions related to physical, emotional and/or financial difficulty, caregivers were asked “Is helping difficult?” If yes, respondents were asked to rate the level of difficulty of helping in each domain on a scale from 1 (“a little difficult”) to 5 (“very difficult”). Following the approach of Vick et al. (2019), difficulty helping was categorized as follows: 0 = no difficulty; 1 = some difficulty (rating of 1, 2 or 3), and 2 = substantial difficulty (4 or 5). During the NSOC, caregivers were also asked to report on the negative aspects of ccaregiving on a 3-point scale (from 0 = not so much to 2 = very much). The items included: (a) exhaustion when the caregiver went to bed at night, (b) having more things to do than they could handle, (c) insufficient free time for themselves, or (d) inability to maintain a routine due to caring for their loved one. We coded positive responses as 1, and negative responses as 0 (Vick et al., 2019).

#### Caregiver Sleep-Related Disturbances

Caregiver sleep-related disturbances were assessed using two separate items. Participants reported how often their sleep was interrupted in the last month because of caregiving using a 5-point scale item (1 = every night, 2 = most nights, 3 = some nights, 4 = rarely, 5 = never) asking, “In the last month, how often did helping the care recipient cause your sleep to be interrupted?” With response options “never”; “rarely”; “some days”; “most days”; “every.” Using similar scale, participants were asked about nighttime awakenings: “In the last month, on nights when you woke up before you wanted to, how often did you have trouble falling back asleep?” We reclassified these responses as “rarely/never”; “some days”; and “most days/every.

#### Caregiver Characteristics

We included several caregiver characteristics in our analyses. Our primary predictor of interest was self-identified Black, Hispanic or white race. Other independent variables included age, gender (male or female), relationship to care recipient [spouse; child (son, daughter, stepdaughter, stepson) / child-in-law (daughter in law, son-in-law or stepson) and other]. We examined assistance with specific task in domains of medical caregiving, disability-related assistance, and personal care. The nursing/medical caregiving domain consisted of eight tasks: keeping track of medications, giving shots or injections, managing medical tasks (e.g., ostomy care, intravenous, helping with exercises, helping with a special diet, caring for skin wounds/sores, caring for teeth/dentures, and caring for feet (1 = yes, 0 = no, score range = 0–4). The mobility domain consisted of four tasks: helping the care recipient get around the house, lifting the care recipient from a seated position, letting the care recipient lean on the caregiver for support, and holding the care recipient when they walked or stood (range 0–4). We also examined whether the caregiver provided assistance with personal care every day, most days, some days, rarely, and never (range 0 to4). Responses to each question (0 = no, 1 = yes) were summed to form a count of the number of tasks for which the caregiver provided assistance within each domain. To assess intensity of help received by PLWD in our sample, we calculated total hours of care received from all caregivers per day. Caregivers reported whether they had been diagnosed with eight health conditions: heart attack, high blood pressure, heart disease, arthritis, diabetes, lung disease, osteoporosis, and stroke (1 = yes, 0 = no). We included an indicator variable for the presence of heart attack, high blood pressure, heart disease, arthritis, osteoporosis and diabetes. We controlled for these variables based on review of prior literature related to caregiver strain or sleep disturbances (Ornstein and Gaugler, 2012; Wolff et al., 2016; Polenick et al., 2017; Stahl et al., 2020).

#### Persons Living With Dementia Characteristics

Persons living with dementia difficulties with sleep initiation and sleep maintenance were assessed with two separate items from the NHATS. These questions were: “In the last month, (1) how often did it take you more than 30 min to fall asleep?” and (2) “on nights when you woke up before you wanted to get up, how often did you have trouble falling back to sleep?” Possible responses to for both questions were: “every night (7 nights/week),” “most nights (5–6 nights/week,” “some nights (2–4 nights/week,” “rarely (<1 nights/week”), and “never.” We reclassified these responses as “rarely/never”; “some days”; and “most days/every.”

### Analysis

We summarized the variables of interest using frequencies (weighted percent). The outcomes of interest were level of caregiving strain (high strain = score of 5 or above), interrupted sleep (rarely/never, some nights, most/every night), and trouble falling back asleep (rarely/never, some nights, most/every night). Predictors of interest obtained from NSOC were caregivers’ age, gender, relationship to PLWD (spouse, adult child, other), presence of each of the following cardiovascular conditions (Heart attack, high blood pressure, heart disease, and diabetes), nature and intensity of care (hours per day), and types of activities for which help is provided across domains of disability-related activities, medical caregiving, and personal care. We also included the following PLWD predictors in the model, age, sleep initiation problem, and sleep maintenance problem obtained from NHATS. We used multivariable logistic regression to analyze the caregiver strain variable. After testing for proportional odds assumption, we used a multivariable proportional odds model to analyze the sleep-related outcomes (trouble falling back to sleep and interrupted sleep).

We analyzed the data using NSOC analytic weight and statistical procedures to account for the complex survey nature of the data. We weighted the analyses to represent the national estimates for non-institutionalized United States caregivers of PLWD. We used Taylor Series linearization for appropriate variance estimates. Our threshold for statistical significance is 0.05. All analyses were performed using SAS 9.4^®^ (Cary, NC, United States). The Johns Hopkins University Institutional Review Board approved the NHATS protocol, and all participants provided written informed consent. The Adelphi University Institutional Review Board deemed this study exempt.

### Study Results

Table 1 provides weighted descriptive statistics for the sample (*n* = 1142), representing 8,721,893 caregivers and 4,733,853 PLWD. Of these caregivers, 64.6% were white, 15% Black, 14% Hispanic and other 2.5%. On average, caregivers were 60 years old (SE = 0.63), and 65.2% were female. The caregivers reported having experienced cardiovascular conditions, with 6% having had a heart attack, 48.3% with high blood pressure, 5.4% with other heart conditions, and 16.7% with diabetes. Over half of the caregivers were the child/stepchild/child-in-law of the care recipient (58.8%). Overall, nearly half of the caregivers reported trouble falling back to sleep (46.8%), 15.4% reported interrupted sleep and 18.4% reported high caregiving strain, measured by a score of 5 or higher on caregiver strain index.

**TABLE 1:** Caregiver and PLWD characteristics (*n* = 1142).

Caregiver characteristics	*N* (%) / Mean (SE)
Age (mean: SE)	60.5 (0.63)
**Gender**	
Male	355 (34.79)
Female	780 (65.21)
**Race/Ethnicity**	
Non-Hispanic White	605 (64.61)
Non-Hispanic Black	370 (14.81)
Hispanic	98 (13.93)
Other	24 (2.47)
Relationship to patient	
Spouse	171 (14.81)
Child/step-child/child-in-law	706 (58.79)
Other	258 (26.4)
**Health-related conditions**	
Heart attack	76 (6.04)
High blood pressure	551 (48.3)
Other heart conditions	72 (5.40)
Diabetes	205 (16.74)
**Characteristics of PLWD**	
**Age**	
65–74	81 (14.62)
75–84	364 (37.45)
≥85	690 (47.93)
**Gender**	
Male 334 (32.76)	
Female	801 (67.24)
**Race/ethnicity**	
Non-Hispanic White	620 (66.3)
Non-Hispanic Black	389 (15.36)
Hispanic	78 (10.71)
Other	20 (3.16)
**Marital status**	
Single/divorced/widowed	813 (65.09)
Married/with partner	322 (34.91)
**Sleep initiation**	
Every night	138 (13.25)
Most nights	174 (14.78)
Some nights	275 (21.47)
Rarely	212 (19.74)
Never	314 (29.03)
**Sleep maintenance**	
Every night	65 (6.77)
Most nights	114 (9.59)
Some nights	335 (27.56)
Rarely	239 (21.17)
Never	323 (28.8)
**Care intensity**	
Hours of help per week (mean: SE)	4.5 (0.18)
**Help getting around**	
Every day	168 (12.5)
Most days	162 (12.75)
Some days	363 (32.38)
Rarely/never	441 (42.36)
**Help with personal care**	
Every day	187 (12.6)
Most days	105 (7.94)
Some days	268 (23.99)
Rarely/never	574 (55.48)
Number of medical/nursing task (mean: SE)	1.95 (0.08)
**Caregiver outcome**	
**Trouble falling back to sleep**	
Every night	49 (4.17)
Most nights	119 (9.95)
Some nights	360 (32.72)
Rarely	331 (27.59)
Never	234 (20.32)
Did not help last month*	20 (2.58)
**Interrupted sleep**	
Every night	20 (1.48)
Most nights	32 (2.17)
Some nights	161 (11.75)
Rarely	257 (20.71)
Never	627 (59.03)
Did not help last month*	20 (2.58)
High strain (composite score ≥ 5)	224 (18.39)

*^a^Data from the 2017 National Health and Aging Trends Study and National Study of Caregiving (unweighted, n = 1142).*

About 86% of the PLWD in the sample were 75 years of age and older with 48% being at least 85 years old. They were mostly female (67.2%) and white (66.3%). About 35% of the PLWD were married or living with a partner. Overall, about 45% reported receiving help with personal care and about 57% received some help with getting around. Nearly half of the PLWD reported that they had trouble falling back to sleep on some nights, most nights, or every night in the last month (44%). Approximately 50% of PLWD reported difficulty with sleep initiation (over 30 min to fall asleep) some nights, most nights, or every night in the last month.

Nearly half of the caregivers reported that they had trouble falling back to sleep on some nights, most nights, or every night in the last month (46.84%). 15.4% of caregivers reported interrupted sleep some nights, most nights, or every night in the last month. Roughly one in five caregivers reported high levels of caregiving strain (18.4%).

### Multivariable Models

#### Factors Associated With Caregiver Sleep-Related Disturbances and Strain

Results from the multivariable regression models are presented in Table 2. In the model for caregiver strain, female caregivers were 2.61 times as likely to report experiencing high caregiver strain compared to male caregivers [OR = 2.61, CI (1.56, 4.39)]. No other variables in the model were significantly associated with high level of strain.

**TABLE 2:** Associations between PLWD and caregiver sleep-related disturbances and strain.

		High level of strain		Sleep interruption		Trouble falling back asleep	
		OR (95% CI)	*P*-value	OR (95% CI)	*P*-value	OR (95% CI)	*P*-value
**Characteristics of caregivers**							
Race/Ethnicity			0.10		0.43		0.03
	Non-hispanic White	Ref	Ref	Ref	Ref	Ref	Ref
	Non-hispanic Black	0.54 (24, 1.21)	0.13	0.89 (0.42, 1.86)	0.75	**0.54 (0.36, 0.82)**	**0.01**
	Hispanic	0.64 (0.29, 1.39)	0.25	0.79 (0.44, 1.43)	0.42	**0.56 (0.34, 0.91)**	**0.02**
Age		0.99 (0.97, 1.01)	0.29	1.00 (0.97, 1.03)	0.84	1.00 (0.98, 1.01)	0.85
Gender							
	Male	Ref	Ref	Ref	Ref	Ref	Ref
	Female	**2.61 (1.56, 4.39)**	**0.001**	0.88 (0.53, 1.48)	0.63	**1.63 (1.12, 2.36)**	**0.01**
Relationship to patient							
	Spouse	Ref	Ref	Ref	Ref	Ref	Ref
	Child/Step-child	1.32 (0.57, 3.06)	0.51	0.49 (0.20, 1.18)	0.11	1.29 (0.68, 2.44)	0.43
	Other	0.35 (0.12, 1.02)	0.06	**0.22 (0.07, 0.71)**	**0.01**	1.06 (0.52, 2.14)	0.87
**Clinical conditions**							
Heart attack		1.36 (0.38, 4.85)	0.63	0.60 (0.18, 1.98)	0.39	0.79 (0.40, 1.56)	0.49
High blood pressure		1.30 (0.86, 1.96)	0.21	1.24 (0.76, 2.01)	0.38	**1.62 (1.12, 2.33)**	**0.01**
Other heart conditions		1.13 (0.31, 4.21)	0.85	0.68 (0.11, 4.11)	0.67	0.72 (0.30, 1.71)	0.45
Diabetes		1.66 (0.85, 3.27)	0.14	0.65 (0.38, 1.11)	0.12	1.11 (0.70, 1.76)	0.65
**Characteristics of PLWD**							
**Age**							
	65-74	Ref	Ref	Ref	Ref	Ref	Ref
	75-84	0.70 (0.30, 1.58)	0.39	1.21 (0.58, 2.54)	0.61	**2.98 (1.83, 4.84)**	**<0.001**
	≥ 85	0.53 (0.30, 1.58)	0.11	1.47 (0.76, 2.82)	0.24	**1.90 (1.13, 3.21)**	**0.02**
**Sleep initiation**							
	Never/Rarely	Ref	Ref	Ref	Ref	Ref	Ref
	Some days	1.87 (0.90, 3.90)	0.09	0.95 (0.46, 1.93)	0.88	1.22 (0.79, 1.89)	0.37
	Most Days/Every Day	1.08 (0.52, 2.23)	0.83	1.30 (0.69, 2.44)	0.41	1.14 (0.73, 1.79)	0.56
**Sleep maintenance**							
	Never/Rarely	Ref	Ref	Ref	Ref	Ref	Ref
	Some days	0.93 (0.45, 1.91)	0.84	1.16 (0.55, 2.42)	0.70	1.21 (0.71, 2.06)	0.47
	Most Days/Every Day	1.76 (0.75, 4.11)	0.19	**2.27 (1.01, 5.10)**	**0.05**	**2.25 (1.39, 3.67)**	**0.002**
**Caregiving intensity**							
Hours of help per day		1.03 (0.99, 1.07)	0.18	**1.07 (1.02, 1.12)**	**0.01**	0.98 (0.95, 1.02)	0.37
**Mobility** (help getting around)							
	Every day	1.29 (0.41, 4.04)	0.66	1.43 (0.61, 3.36)	0.40	0.79 (0.43, 1.47)	0.45
	Most days	2.19 (0.92, 5.22)	0.07	2.17 (0.98, 4.79)	0.06	1.03 (0.60, 1.76)	0.91
	Some days	1.16 (0.62, 2.18)	0.63	1.37 (0.72, 2.61)	0.34	1.01 (0.68, 1.49)	0.96
	Rarely/Never	Ref	Ref	Ref	Ref	Ref	Ref
**Help with personal care**							
	Every day	2.46 (0.99, 6.10)	0.05	**7.71 (3.64, 16.34)**	**<0.001**	**2.37 (1.20, 4.68)**	**0.01**
	Most days	1.22 (0.43, 3.46)	0.71	**2.67 (1.13, 6.29)**	**0.03**	1.57 (0.75, 3.31)	0.23
	Some days	1.51 (0.85, 2.69)	0.16	**2.07 (1.14, 3.78)**	**0.02**	1.18 (0.77, 1.81)	0.44
	Rarely/Never	Ref	Ref	Ref	Ref	Ref	Ref
**Number of nursing/Medical caregiving tasks**		1.07 (0.99, 1.07)	0.31	1.09 (0.94, 1.26)	0.25	1.05 (0.93, 1.19)	0.45

In the model for interrupted sleep and trouble falling back to sleep, compared to the spouses who are caregivers, caregivers who were of “other” relationships were less likely to report higher levels of sleep interruption [OR = 0.22, CI (0.07, 0.71)]. Each unit increase in the number of daily hours spent in caregiving was associated with a corresponding increase in the odds of sleep interruption in the caregiver [OR = 1.07, CI (1.01, 1.12)]. Caregivers who provided PLWDs with help getting around most days reported greater sleep interruptions than those who rarely/never help getting around [OR = 2.27, 95% CI (1.01, 5.10)]. Higher frequency of assistance with personal care was associated with greater sleep interruption. Caregivers who help PLWD with personal care every day, most days and some days had greater odds of reporting higher level of sleep interruption compared to those who help with personal care rarely or never [OR = 7.71, CI (3.64, 16.34), OR = 2.67, CI (1.13, 6.29), and OR = 2.07, CI (1.14, 3.78), respectively].

Caregivers of PLWD 75–84 and 85 and older compared to 65–74 had greater odds of reporting higher level of trouble falling back asleep [OR = 2.98 CI (1.83, 4.84) and OR = 1.90 CI (1.13, 3.21)]. The odds of reporting greater trouble falling back asleep was significantly greater among female (vs. male) caregivers [OR = 1.63, CI (1.12, 2.36)]. Though the other medical factors were not significant, the odds of reporting greater trouble falling back asleep was significantly greater among caregivers with high blood pressure vs. caregivers without high blood pressure [OR = 1.62, CI (1.12, 2.33)]. Caregivers who help PLWD with personal care every day had increased odds of reporting higher trouble falling back asleep compared to those who help with personal care rarely or never [OR = 2.37, CI (1.20, 4.68)].

Compared to non-Hispanic Whites, non-Hispanic Blacks and Hispanics caregivers had reduced odd of reporting greater trouble falling back asleep [OR = 0.55, CI (0.36, 0.82) and OR = 0.56, CI (0.34, 0.91), respectively].

#### Association Between Persons Living With Dementia Characteristics and Caregiver Sleep-Related Disturbances

Persons living with dementia’s who reported trouble falling back asleep most days/every day resulted in greater odds of caregiver’s high level of interrupted sleep (OR = 2.25, 95% CI = 1.39, 3.67) compared to PLWD who never/rarely have sleep reported trouble falling back asleep.

## Discussion

This study investigated predictors of sleep disturbance and caregiver burden among family caregivers of PLWD, using data from a nationally representative sample of Medicare beneficiaries. Our analysis yielded two key findings. First, compared to non-Hispanic Whites, non-Hispanic Black and Hispanic caregivers had reduced odds of reporting greater trouble falling back asleep. Second, the odds of reporting greater trouble falling back asleep was significantly higher among caregivers with high blood pressure vs. caregivers without high blood pressure. Third, we found no racial/ethnic differences in interrupted sleep or caregiver strain. Our findings extend the caregiver literature by specifically identifying high blood pressure as a significant correlate of inadequate sleep (trouble falling back asleep) among family caregivers of PLWD using nationally representative data. Inadequate sleep is a modifiable lifestyle component essential to ameliorate cardiometabolic risk factors, including blood pressure (St-Onge et al., 2016). Our results add to the accumulating evidence suggesting that caregivers of PLWD commonly experience sleep problems, which are often linked to the caregiver’s own health and risk for subsequent adverse health outcomes (Adelman et al., 2014; Leggett et al., 2018).

The findings of a strong association between greater trouble falling back asleep and high blood pressure are noteworthy given the high prevalence of vascular contributors (e.g., hypertension) to risk of dementia. The direction of the relationship, whether high blood pressure predicts trouble falling back asleep or trouble falling back asleep predicts high blood pressure, was unable to be examined because the current analysis was cross-sectional. Yet, prior research strongly suggest that mid-life high blood pressure increases the risk of late-life cognitive impairment (Elias et al., 2012). Sleep fragmentation or arousal from sleep is known to increase systemic blood pressure (Morrell et al., 2000), which is worse for Blacks (Williams et al., 2016; Yano et al., 2020).

Interestingly, we observed no significant racial/ethnic differences in trouble falling back asleep sleep or caregiver strain. Our findings of greater association of self-reported sleep disturbances (trouble falling back asleep) among white caregivers compared to Black caregivers is inconsistent with most published longitudinal and cross-sectional studies that have shown that individuals of the Black race are characterized by a greater prevalence of inadequate sleep (Stamatakis et al., 2007; Jean-Louis et al., 2015; Williams et al., 2016). There are several plausible explanations for the association we observed. Other studies have found that, compared to white women, minoritized women, despite reporting less adequate sleep, are less likely to report trouble sleeping, providing evidence of an important health disparity (Amyx et al., 2017). For example, caregiver stress or role overload may drive the association between trouble falling back asleep and high blood pressure (Adelman et al., 2014; Liang et al., 2020). Furthermore, it is possible that different racial/ethnic factors may affect minority caregivers’ self-perceived level of the burden of their caregiving role. For example, prior research has shown that Black caregivers are more likely to report emotional gains and less likely to report emotional difficulty than whites (Beach et al., 2019; Fabius et al., 2020; Liu et al., 2020). For example multiple studies have found that United States-born Hispanic/Latina, Chinese, and Japanese immigrants were more likely to report sleep complaints than their first-generation ethnic counterparts, a finding largely explained by language acculturation and unmeasured factors associated with language acculturation (Hale et al., 2014; Jackson et al., 2014; Cunningham et al., 2016).

Beyond our findings about racial difference among caregivers of PLWD, other results merit further clarification. Our finding that female caregivers were more likely to report experiencing high caregiver strain and trouble falling back asleep compared to male caregivers is consistent with prior studies (Vick et al., 2019) and reflects the predominance of women as dementia caregivers (Riffin et al., 2017; Vick et al., 2019). Similarly, our finding that higher caregiving intensity (daily hours spent caregiving, help with personal care and mobility every day or most days) was associated with greater odds of interrupted sleep and trouble falling back asleep was unsurprising and in line with prior literature (Polenick et al., 2017; Leggett et al., 2018), and include an association with daily assistance with personal care and mobility.

Given the growing population of PLWD living in the community, with heavy reliance on family caregivers, our study has important implications. The health consequences associated with poor sleep for caregivers of PLWD underscore the need for structured support systems for this population. Strategies such as standardized caregiver assessment tools for determination of caregiver ability and risk factors for conditions such as high blood pressure, are warranted to support the caregiver’s health, and ultimately improve outcomes of the PLWD (Riffin et al., 2021). This could help identify caregivers in whom interventions aimed at reducing sleep disturbances should be targeted.

Strengths of this study include a nationally representative sample of United States caregivers to PLWD, and the investigation of racial differences associated with caregiver sleep and strain, given the project increase in the minority PLWD. However, this study has limitations. First, sleep disturbances were measured by self-report, rather than objective measurement of sleep captured by polysomnography or actigraphy. Research using validated measures of sleep such as the Pittsburgh Sleep Quality Index (Buysse et al., 1989) are needed to corroborate findings based on subjective data. Additionally, the cross-sectional observational study design precludes making causal inferences between sleep measures and high blood pressure. Future longitudinal studies that include validated measures of sleep quality (Buysse et al., 1989) are warranted to examine these associations that may suggest causal links.

## Conclusion

In summary, the present research adds to current literature by identifying predictors of sleep disturbance and strain among family caregivers who actively support PLWD in the community. Our study findings suggest there is a cross-sectional association between greater trouble falling back asleep and high blood pressure. Further study is warranted to examine if this relationship persists longitudinally and to examine the direction of the association. Our findings suggest that caregivers to PLWD may be at risk for poor health and caregiving outcomes from care-related sleep disturbances. Clearly, there is a need for effective interventions aimed at caregivers of PLWD, a population already susceptible to poor health outcomes.

## Data Availability Statement

The data analyzed in this study is subject to the following licenses/restrictions: NHATS NSOC Sensitive Data. Requests to access these datasets should be directed to https://nhats.org/sites/default/files/inline-files/Obtaining_NHATS_Sensitive_Data_Files_v13_0.pdf.

## Ethics Statement

The studies involving human participants were reviewed and approved by Adelphi University Institutional Review Board. The patients/participants provided their written informed consent to participate in this study. The NHATS research team conducted the interviews and obtained written informed consent.

## Author Contributions

ZO and GJ-L: conceptualization. ZO, RSF-C, and GJ-L: methodology. RSF-C: formal analysis and data curation. ZO, CS, and RSF-C: writing—original draft preparation. ZO, RSF-C, CS, OB, CO, AT, ST, and GJ-L: writing—review and editing. All authors have read and agreed to the published version of the manuscript.

## Conflict of Interest

The authors declare that the research was conducted in the absence of any commercial or financial relationships that could be construed as a potential conflict of interest.

## Publisher’s Note

All claims expressed in this article are solely those of the authors and do not necessarily represent those of their affiliated organizations, or those of the publisher, the editors and the reviewers. Any product that may be evaluated in this article, or claim that may be made by its manufacturer, is not guaranteed or endorsed by the publisher.

## References

[B1] Adelman R. D. Tmanova L. L. Delgado D. Dion S. Lachs M. S. (2014). Caregiver burden: a clinical review. *JAMA* 311 1052–1060. 24618967 10.1001/jama.2014.304

[B2] Alzheimers Association (2019). *Alzheimer’s Disease Facts And Figures.* Hoboken, NJ: John Wiley & Sons.

[B3] Amjad H. Roth D. L. Samus Q. M. Yasar S. Wolff J. L. (2016). Potentially unsafe activities and living conditions of older adults with dementia. *J. Am. Geriatr. Soc.* 64 1223–1232. 10.1111/jgs.14164 27253366 PMC4914464

[B4] Amyx M. Xiong X. Xie Y. Buekens P. (2017). Racial/Ethnic differences in sleep disorders and reporting of trouble sleeping among women of childbearing age in the United States. *Matern. Child Health J.* 21 306–314. 10.1007/s10995-016-2115-9 27439422 PMC5250592

[B5] Badana A. N. S. Marino V. Haley W. E. (2019). Racial differences in caregiving: variation by relationship type and dementia care status. *J Aging Health* 31 925–946. 10.1177/0898264317743611 29254432

[B6] Beach S. R. Kinnee E. Schulz R. (2019). Caregiving and place: combining geographic information system (gis) and survey methods to examine neighborhood context and caregiver outcomes. *Innov. Aging* 3:igz025. 10.1093/geroni/igz025 31528713 PMC6735773

[B7] Black B. S. Johnston D. Leoutsakos J. Reuland M. Kelly J. Amjad H. (2019). Unmet needs in community-living persons with dementia are common, often non-medical and related to patient and caregiver characteristics. *Int. Psychogeriatr.* 31 1643–1654. 10.1017/S1041610218002296 30714564 PMC6679825

[B8] Brodaty H. Woodward M. Boundy K. Ames D. Balshaw R. (2014). Prevalence and predictors of burden in caregivers of people with dementia. *Am. J. Geriatr. Psychiatry* 22 756–765. 10.1016/j.jagp.2013.05.004 24012226

[B9] Burgdorf J. G. Arbaje A. I. Wolff J. L. (2020). Training needs among family caregivers assisting during home health, as identified by home health clinicians. *J. Am. Med. Dir. Assoc.* 21 1914–1919. 10.1016/j.jamda.2020.05.032 32641271 PMC7708398

[B10] Buysse D. Reynolds C. Monk T. Berman S. R. C. F. I. I. I. Kupfer D. J. (1989). The pittsburgh sleep quality index: a new instrument for psychiatric practice and research psychiatry. *Research* 28 193–213. 10.1016/0165-1781(89)90047-4 2748771

[B11] Cook S. K. Cohen S. A. (2018). Sociodemographic disparities in adult child informal caregiving intensity in the United States: results from the new national study of caregiving. *J. Gerontol. Nurs.* 44 15–20. 10.3928/00989134-20180808-05 30148528

[B12] Cunningham T. J. Wheaton A. G. Ford E. S. Croft J. B. (2016). Racial/ethnic disparities in self-reported short sleep duration among US-born and foreign-born adults. *Ethn. Health* 21 628–638. 10.1080/13557858.2016.1179724 27150351 PMC5206750

[B13] DeMatteis J. Freedman V. Kasper J. (2016). *National Health and Aging Trends Study Round 5 Sample Design And Selection.* Baltimore, MD: Johns Hopkins University School of Public Health.

[B14] Donaldson C. Tarrier N. Burns A. (1998). Determinants of carer stress in Alzheimer’s disease. *Int. J. Geriatr. Psychiatry* 13 248–256. 10.1002/(sici)1099-1166(199804)13:4<248::aid-gps770>3.0.co;2-0 9646153

[B15] Elias M. F. Goodell A. L. Dore G. A. (2012). Hypertension and cognitive functioning: a perspective in historical context. *Hypertension* 60 260–268. 10.1161/HYPERTENSIONAHA.111.186429 22753214

[B16] Fabius C. D. Wolff J. L. Kasper J. D. (2020). Race differences in characteristics and experiences of black and white caregivers of older americans. *Gerontologist* 60 1244–1253. 10.1093/geront/gnaa042 32400881 PMC7491434

[B17] Freedman V. A. Spillman B. C. (2014). Disability and care needs among older Americans. *Milbank Q.* 92 509–541. 10.1111/1468-0009.12076 25199898 PMC4221755

[B18] Galvin J. Roe C. Powlishta K. Coats M. A. Muich S. J. Grant E. (2005). The AD8: a brief informant interview to detect dementia. *Neurology* 65 559–564. 16116116 10.1212/01.wnl.0000172958.95282.2a

[B19] Gao C. Chapagain N. Y. Scullin M. K. (2019). Sleep duration and sleep quality in caregivers of patients with dementia: a systematic review and meta-analysis. *JAMA Network Open* 2:e199891. 10.1001/jamanetworkopen.2019.9891 31441938 PMC6714015

[B20] Gehrman P. Gooneratne N. S. Brewster G. S. Richards K. C. Karlawish J. (2018). Impact of Alzheimer disease patients’ sleep disturbances on their caregivers. *Geriatr. Nurs.* 39 60–65. 10.1016/j.gerinurse.2017.06.003 28684102 PMC5752633

[B21] Hale L. Troxel W. M. Kravitz H. M. Hall M. H. Matthews K. A. (2014). Acculturation and sleep among a multiethnic sample of women: the Study of Women’s Health Across the Nation (SWAN). *Sleep* 37 309–317. 10.5665/sleep.3404 24497659 PMC3900614

[B22] Hebert L. E. Weuve J. Scherr P. A. Evans D. A. (2013). Alzheimer disease in the United States (2010–2050) estimated using the 2010 census. *Neurology* 80 1778–1783. 10.1212/WNL.0b013e31828726f5 23390181 PMC3719424

[B23] Jackson C. L. Hu F. B. Redline S. Williams D. R. Mattei J. Kawachi I. (2014). Racial/ethnic disparities in short sleep duration by occupation: the contribution of immigrant status. *Soc. Sci. Med.* 118 71–79. 10.1016/j.socscimed.2014.07.059 25108693 PMC4744798

[B24] Jean-Louis G. Grandner M. A. Youngstedt S. D. Williams N. J. Zizi F. Sarpong D. F. (2015). Differential increase in prevalence estimates of inadequate sleep among black and white Americans. *BMC Public Health* 15:1185. 10.1186/s12889-015-2500-0 26611643 PMC4661980

[B25] Jennings L. A. Reuben D. B. Evertson L. C. Serrano K. S. Ercoli L. Grill J. (2015). Unmet needs of caregivers of individuals referred to a dementia care program. *J. Am. Geriatr. Soc.* 63 282–289. 10.1111/jgs.13251 25688604 PMC4332558

[B26] Kasper J. D. Freedman V. A. Spillman B.C. (2013). *Classification of Persons by Dementia Status in the National Health and Aging Trends Study. Technical Paper #5.* Baltimore, MD: Johns Hopkins University School of Public Health.

[B27] Kasper J. D. Freedman V. A. Spillman B. C. Wolff J. L. (2015). The disproportionate impact of dementia on family and unpaid caregiving to older adults. *Health Aff. (Millwood).* 34 1642–1649. 10.1377/hlthaff.2015.0536 26438739 PMC4635557

[B28] Leggett A. Polenick C. A. Maust D. T. Kales H. C. (2018). “What hath night to do with sleep?”: the caregiving context and dementia caregivers’, Nighttime Awakenings. *Clin. Gerontol.* 41 158–166. 10.1080/07317115.2017.1352057 28967849 PMC6075725

[B29] Liang J. Aranda M. P. Lloyd D. A. (2020). Association between role overload and sleep disturbance among dementia caregivers: the impact of social support and social engagement. *J. Aging Health* 32 1345–1354. 10.1177/0898264320926062 32524886

[B30] Liu C. Badana A. N. S. Burgdorf J. Fabius C. D. Roth D. L. Haley W. E. (2020). Systematic review and meta-analysis of racial and ethnic differences in dementia caregivers’ well-being. *Gerontologist* 61 e228–e243. 10.1093/geront/gnaa028 32271380 PMC8276619

[B31] Mather M. A. Laws H. B. Dixon J. S. Ready R. E. Akerstedt A. M. (2020). Sleep behaviors in persons with alzheimer’s disease: associations with caregiver sleep and affect. *J. Appl. Gerontol.* 41 295–305. 10.1177/0733464820979244 33353457

[B32] McCurry S. M. Logsdon R. G. Teri L. Gibbons L. E. Kukull W. A. Bowen J. D. (1999). Characteristics of sleep disturbance in community-dwelling Alzheimer’s disease patients. *J. Geriatr. Psychiatry Neurol.* 12 53–59. 10.1177/089198879901200203 10483925

[B33] Mills P. J. Ancoli-Israel S. von Känel R. Mausbach B. T. Aschbacher K. Patterson T. L. (2009). Effects of gender and dementia severity on Alzheimer’s disease caregivers’ sleep and biomarkers of coagulation and inflammation. *Brain Behav. Immun.* 23 605–610. 10.1016/j.bbi.2008.09.014 18930805 PMC2757046

[B34] Montaquila J. F. V. Edwards B. Kasper J. (2012). *NHATS Technical Paper #1. 2012; National Health and Aging Trends Study round 1 Sample Design And 1selection.* Baltimore, MD: Johns Hopkins University School of Public Health

[B35] Morrell M. J. Finn L. Kim H. Peppard P. E. Safwan Badr M. Young T. (2000). Sleep fragmentation, awake blood pressure, and sleep-disordered breathing in a population-based study. *Am. J. Respir. Crit. Care Med.* 162 2091–2096. 10.1164/ajrccm.162.6.9904008 11112120

[B36] Ornstein K. Gaugler J. E. (2012). The problem with “problem behaviors”: a systematic review of the association between individual patient behavioral and psychological symptoms and caregiver depression and burden within the dementia patient-caregiver dyad. *Int. Psychogeriatr.* 24 1536–1552. 10.1017/S1041610212000737 22612881 PMC5769475

[B37] Ownby R. L. Saeed M. Wohlgemuth W. Capasso R. Acevedo A. Peruyera G. (2010). Caregiver reports of sleep problems in non-hispanic white, hispanic, and african american patients with Alzheimer dementia. *J. Clin. Sleep Med.* 6 281–289. 20572423 PMC2883041

[B38] Polenick C. A. Leggett A. N. Kales H. C. (2017). Medical care activities among spouses of older adults with functional disability: implications for caregiving difficulties and gains. *Am. J. Geriatr. Psychiatry.* 25 1085–1093. 10.1016/j.jagp.2017.05.001 28652082 PMC5650913

[B39] Reinhard S. C. Feinberg L. F. Choula R. Evans M. (2019). *Valuing the Invaluable: 2019 Update. Charting a Path Forward.* Washington, DC: Aarp Public Policy Institute.

[B40] Riffin C. Van Ness P. H. Wolff J. L. Fried T. (2017). Family and other unpaid caregivers and older adults with and without dementia and disability. *J. Am. Geriatr. Soc.* 65 1821–1828. 10.1111/jgs.14910 28426910 PMC5555780

[B41] Riffin C. Wolff J. L. Pillemer K. A. (2021). Assessing and addressing family caregivers. Needs and risks in primary care. *J. Am. Geriatr. Soc.* 69 432–440. 10.1111/jgs.16945 33217776 PMC8062767

[B42] Rose K. M. Beck C. Tsai P. F. Liem P. H. Davila D. G. Kleban M. (2011). Sleep disturbances and nocturnal agitation behaviors in older adults with dementia. *Sleep* 34 779–786. 10.5665/SLEEP.1048 21629366 PMC3098946

[B43] Roth D. L. Brown S. L. Rhodes J. D. Haley W. E. (2018). Reduced mortality rates among caregivers: does family caregiving provide a stress-buffering effect? *Psychol. Aging.* 33 619–629. 10.1037/pag0000224 29723004 PMC6002922

[B44] Roth D. L. Fredman L. Haley W. E. (2015). Informal caregiving and its impact on health: a reappraisal from population-based studies. *Gerontologist* 55 309–319. 10.1093/geront/gnu177 26035608 PMC6584119

[B45] Schulz R. Martire L. M. (2004). Family caregiving of persons with dementia: prevalence, health effects, and support strategies. *Am. J. Geriatr. Psychiatry* 12 240–249. 15126224

[B46] Skolarus L. E. Freedman V. A. Feng C. Burke J. F. (2017). African american stroke survivors: more caregiving time, but less caregiving burden. *Circ. Cardiovasc. Qual. Outcomes* 10:e003160. 10.1161/CIRCOUTCOMES.116.003160 28228451 PMC5325085

[B47] Stahl S. T. Rodakowski J. Smagula S. F. (2020). Timing of daily activities over a 24-hour period and affective status among a national cohort of older dementia caregivers. *J. Aging Health* 33 125–132. 10.1177/0898264320962363 32975475 PMC7856063

[B48] Stamatakis K. A. Kaplan G. A. Roberts R. E. (2007). Short sleep duration across income, education, and race/ethnic groups: population prevalence and growing disparities during 34 years of follow-up. *Ann. Epidemiol.* 17 948–955. 10.1016/j.annepidem.2007.07.096 17855122 PMC2140008

[B49] St-Onge M. P. Grandner M. A. Brown D. Conroy M. B. Jean-Louis G. Coons M. (2016). Sleep duration and quality: impact on lifestyle behaviors and cardiometabolic health: a scientific statement from the american heart association. *Circulation* 134 e367–e386. 10.1161/CIR.0000000000000444 27647451 PMC5567876

[B50] Tractenberg R. E. Singer C. M. Kaye J. A. (2005). Symptoms of sleep disturbance in persons with Alzheimer’s disease and normal elderly. *J. Sleep Res.* 14 177–185. 10.1111/j.1365-2869.2005.00445.x 15910515 PMC4371732

[B51] Vick J. B. Ornstein K. A. Szanton S. L. Dy S. M. Wolff J. L. (2019). Does caregiving strain increase as patients with and without dementia approach the end of life? *J. Pain Symp. Manag.* 57 199–208. 10.1016/j.jpainsymman.2018.11.004 30453054 PMC6348024

[B52] Williams N. J. Grandner M. A. Wallace D. M. Cuffee Y. Airhihenbuwa C. Okuyemi K. (2016). Social and behavioral predictors of insufficient sleep among African Americans and Caucasians. *Sleep Med.* 18 103–107. 10.1016/j.sleep.2015.02.533 26514614 PMC5070606

[B53] Wolff J. L. Mulcahy J. Huang J. Roth D. L. Covinsky K. Kasper J. D. (2018). Family caregivers of older adults, 1999–2015: trends in characteristics, circumstances, and role-related appraisal. *Gerontologist* 58 1021–1032. 10.1093/geront/gnx093 28637266 PMC6215459

[B54] Wolff J. L. Spillman B. C. Freedman V. A. Kasper J. D. (2016). A national profile of family and unpaid caregivers who assist older adults with health care activities. *JAMA Int. Med.* 176 372–379. 10.1001/jamainternmed.2015.7664 26882031 PMC4802361

[B55] Yano Y. Gao Y. Johnson D. A. Carnethon M. Correa A. Mittleman M. A. (2020). Sleep characteristics and measures of glucose metabolism in blacks: the Jackson heart study. *J. Am. Heart Assoc.* 9:e013209. 10.1161/JAHA.119.013209 PMC7428566 32342760

